# Supplementary material for: From sequence to enzyme mechanism using multi-label machine learning
Source: BMC Bioinformatics. 2014 May 19;15:150. doi: 10.1186/1471-2105-15-150 (PMC4229970; doi:10.1186/1471-2105-15-150)
Supplement: Additional file 2 — Java code of ml2db. Additional file ml2db_code.tar.gz contains the Java source code to run the multi-label machine learning experiments and save the results to database. The code’s Javadoc is included. [file 1471-2105-15-150-S2.zip › additional file 2/ml2db/ml2db/doc/index-files/index-23.html]

W-Index


JavaScript is disabled on your browser.


- Overview
- Package
- Class
- Use
- Tree
- Deprecated
- Index
- Help

- Prev Letter
- Next Letter

- Frames
- No Frames

- All Classes

A B C D E F G H I J K L M N O P Q R S T U V W X 


## W

WEB\_PAGE - Static variable in class main.ml2db.ProjectParameters


WekaTest - Class in test.ml2db.weka
:   Class to test basic weka instance creation

WekaTest() - Constructor for class test.ml2db.weka.WekaTest


write() - Method in class main.ml2db.weka.DataSetWriter


writeArff(boolean, boolean) - Method in class main.ml2db.weka.Arff


writeArffRecordToDb(Properties) - Method in class main.ml2db.weka.ArffRecordsTable


writeAttributesAndClasses(Instances) - Method in class main.ml2db.weka.DataSetWriter
:   Write the data instances to file

writeBaseArff() - Method in class main.ml2db.weka.DataSetManager


writeDataStatistics() - Method in class main.ml2db.weka.DataSetWriter
:   Logs to file the number of instances, classes and attributes in the
    dataset.

writeDotSourceToFile(String) - Method in class main.ml2db.graphviz.GraphViz
:   Writes the source of the graph in a file, and returns the written file as
    a File object.

writeFiles() - Method in class main.ml2db.mulan.attributesfiltered.AttFilter


writeGraphToFile(byte[], File) - Method in class main.ml2db.graphviz.GraphViz
:   Writes the graph's image in a file.

writeGraphToFile(byte[], String) - Method in class main.ml2db.graphviz.GraphViz
:   Writes the graph's image in a file.

writeHeader() - Method in class main.ml2db.weka.DataSetWriter


writeInstanceToFile(String, Instance, String) - Method in class main.ml2db.weka.DataSetWriter
:   Write an individual Weka instance to file

writeObjectOverride(Object) - Method in class main.ml2db.DebuggingObjectOutputStream


writeTail() - Method in class main.ml2db.weka.DataSetWriter

A B C D E F G H I J K L M N O P Q R S T U V W X

- Overview
- Package
- Class
- Use
- Tree
- Deprecated
- Index
- Help

- Prev Letter
- Next Letter

- Frames
- No Frames

- All Classes
